# In Honor of John Bannister Goodenough, an Outstanding Visionary

**DOI:** 10.3390/molecules26216624

**Published:** 2021-11-01

**Authors:** Jean Etourneau, Claude Delmas, Stéphane Jobic, Myung-Hwan Whangbo

**Affiliations:** 1 ICMCB-CNRS, University of Bordeaux, 33600 Pessac, France; delmas@icmcb-bordeaux.cnrs.fr; 2 Institut des Matériaux Jean Rouxel, Université de Nantes, CNRS, IMN, 44000 Nantes, France; stephane.jobic@cnrs-imn.fr; 3 Department of Chemistry, North Carolina State University, Raleigh, NC 27695, USA

John B. Goodenough won the Nobel Prize in Chemistry in 2019 with Stanley Wittingham and Akira Yoshino for their fundamental contributions to the development of lithium-ion batteries. Calls for recognition for John’s pioneering work on lithium-ion batteries had been launched at the Nobel Committee for many years and finally the calls were answered! This was wonderful, though long overdue, news for the communities of solid-state chemistry and materials sciences. John impacted these areas of research greatly via his constant desire to account for the physical properties of solids through the discussion of their local crystal structures and their chemical bonding. Let us notice that, on this special occasion, John also set the record for being the oldest person, at 97, to receive this prestigious honor!

John is a world-renowned outstanding scientist who won the Nobel Prize not only for his pioneering work on lithium-ion batteries, but also for his profound influence on solid-state chemistry, paving the way into the 21st century toward the modern approach to materials sciences where physics and chemistry are intimately related. Very early in the sixties of the last century, he demystified solid-state physics for solid-state chemists by developing qualitative tools with which to discuss and predict in two areas of transition-metal oxides: (i) he showed how to understand the magnetism of transition-metal oxides in terms of spin exchanges between transition-metal magnetic ions bridged by a common main-group ligand atom, providing the qualitative rules known as the Goodenough–Kanamori rules, and (ii) he related the metal versus insulator behaviors of transition-metal oxides to whether the interactions between adjacent transition-metal atoms dominate over those involving individual transition-metal atoms. In both areas of research, John showed how the seemingly complex problems can be reduced to the level of chemical bonding and local structure–property relationships, thereby strongly inspiring generations of scientists!

During the International Conference organized by Paul Hagenmuller in Bordeaux in 1964, which gathered the most influential solid-state physicists and chemists from Europe and North America involved in the study of transition-metal oxides, John clearly demonstrated the importance of solid-state physics in understanding the physical properties of solids with a view to using them for applications. From that moment on, John became a mentor and a guide for many of us in the discipline of solid-state chemistry. Throughout his stays in Europe and Bordeaux in particular, John inspired people around him by his vision and his fruitful intuition. John was a pioneer not only in lithium-ion batteries but also in developing phenomenological mechanisms useful in explaining a variety of physical properties of transition-metal oxides. For further discussion, see the article of M. Pouchard [1].

John Goodenough is one of those rare scientists whose impact on our daily lives is not only obvious, but also essential. His contributions have appreciably changed the way we live and, in a world always in search of new and better energy solutions, point towards promising directions for the future. Even at the age of 99, he still continues to develop new polymers and battery concepts with researchers in his laboratory. He is now largely focused on developing fully solid-state batteries with many challenging requirements such as low cost, long cycle life, high volumetric density and fast rates of charge and discharge.

John is probably one of first to establish a strong link between basic and applied research, which is of prime importance for our society. John showed an insatiable desire to interpret observations of extended solid-state compounds by developing conceptual tools based on local bonding and local structures. With this Special Issue dedicated to John Goodenough, we salute him for his long and illustrious career, significantly advancing the discipline of solid-state chemistry and materials sciences. For all of us, John is clearly an example to follow.

John Goodenough has a warm personality and he often said that, to live one’s life to the fullest, one should be able to interact with people who want to interact with you. All our discussions with John, professional or private (see in Figure 1), were extremely rich in a friendly and relaxed atmosphere punctuated by his legendary and contagious laughter! John enjoyed traveling all over the world and sharing scientific discussions with people from almost every country in the world.

## 1. Special Issue Dedicated to Professor John B. Goodenough

After the introduction by Michel Pouchard [1] that spotlights the outstanding contributions of Professor John B. Goodenough to solid-state chemistry, this Special Issue consists of 14 contributions representing two areas of research John worked on, namely, nine contributions on lithium-ion batteries and five on magnetic properties.

From a theory describing the physical properties of a system, we often demand two things that are often incompatible with each other. One is to demand a conceptual framework with which to organize what is observed and predict qualitative trends. The other is to demand a quantitative tool with which to describe with numerical accuracy. The Goodenough–Kanamori rules, formulated in the late 1950s, provided a conceptual framework with which to discuss whether spin exchange interactions between transition-metal magnetic ions M present in M-L-M bridges, formed by sharing a common main group ligand atom L, are antiferromagnetic or ferromagnetic. The conceptual picture given by the Goodenough–Kanamori rules has greatly influenced the thinking of chemists and physicists for many decades. 

The 2019 Nobel Prize in chemistry to John B. Goodenough, underlines another important scientific field of interest of John, namely, the development of lithium-ion batteries that are now indispensable in our lives (smartphones, laptops, electric vehicles, etc.). Goodenough was particularly credited for the choice of Li_x_CoO_2_ and then Li_x_NiO_2_ materials as positive electrodes in batteries, which truly revolutionized the field of electrochemistry. He also showed that the potential delivered by a battery can be increased by replacing oxide anions by polyanions (LiFePO_4_), and continues to develop solid electrolytes with high conductivity of ionic species. All these materials or their derivatives are now used in commercial batteries.

In what follows, we briefly comment on the main points of each contribution.

### 1.1. Contributions in the Area of Spin Exchanges and Magnetism

In the following, the first four papers are invited contributions.

(1) In the late 1990s and early 2000s, it became increasingly clear that spin exchange interactions between magnetic ions do occur even if they do not share a common ligand. They are M-L…L-M and M-L…A…L-M type interactions with A as a d^0^ (i.e., S = 0) cation. They reflect that the magnetic orbitals of a cation M forming an ML_n_ polyhedron with surrounding ligands are given by d-states of ML_n_, in which the p-orbitals of L are combined out-of-phase with the d-orbitals of M, and that all types of spin exchanges are governed largely by the ligand p-orbitals of the magnetic orbitals. The qualitative aspects of spin exchanges were reviewed by Myung-Hwan Whangbo, Hyun-Joo Koo and Reinhard K. Kremer [2], in which they discuss how the qualitative trends in spin exchanges are related to the arrangements of the magnetic orbitals, providing the structure–property relations with which to understand the magnetic properties of complex magnetic solids.

(2) Interactions between spins of a magnetic system are very weak and are very small in energy scale. This necessitates the use of a spin Hamiltonian defined in terms of several phenomenological parameters (e.g., spin exchanges, Dzyaloshinskii–Moriya interactions, etc.) to have quantitative predictions on magnetic properties. How to estimate the numerical values of such parameters systematically and accurately has been a challenge for a long time, which eventually led to an energy-mapping analysis based on first-principles DFT+U and DFT+hybrid calculations in the early to mid-2000s. To determine the parameters defining a model spin Hamiltonian, one analyzes the relative energies of a set of broken-symmetry ordered spin states using two different Hamiltonians; one generates the relative energies by using the model Hamiltonian made up of the parameters to determine and also by performing DFT+U or DFT+hybrid numerical calculations. Then, the two energy spectra are mapped to determine the numerical values of the desired parameters. This energy-mapping analysis is generalized in the review written by Xueyang Li, Hongyu Yu, Feng Lou, Junsheng Feng, Myung-Hwan Whangbo and Hongjun Xiang [3]. This review examines the origin of various possible interactions and shows how to compute the values of these interactions. Having such a quantitative tool is crucial because the purpose of using a model spin Hamiltonian is not to include all possible phenomenological parameters, but to include the minimal number of parameters needed to describe the observed physical properties. The quantitative tool allows one to sort out which parameters are essential. 

(3) The spin exchange parameters can be estimated by using a method different from the energy-mapping method. For instance, when non-spin-polarized DFT calculations are employed to describe a magnetic solid of transition-metal magnetic ions, there occur partially filled d-state band(s) so that the solid is predicted to be metallic instead of magnetic insulating. Nevertheless, one can determine the parameters of a tight binding approximation that can reproduce the calculated d-state electronic band structure. From such parameters, the spin exchange parameters necessary for discussing the magnetic properties can be determined. Tanusri Saha-Dasgupta analyzed the d-state band structures in terms of the N-th order muffin orbital-downfolding technique to determine the spin lattices appropriate for a number of insulating magnetic oxides. Saha-Dasgupta provided a review on her studies based on this method in [4]. 

(4) In each layer of a layered compound MPS_3_ (M = Mn, Fe, Co, Ni), the metal ions M^2+^ form a honeycomb arrangement with a molecular anion P_2_S_6_^4−^ located at the center of every hexagon of M^2+^ ions. Thus, the spin exchange between M^2+^ ions is mediated by the symmetry-adapted group orbitals of P_2_S_6_^4−^. Hyun-Joo Koo, Reinhard K. Kremer and Myung-Hwan Whangbo [5] probed the spin exchanges of MPS_3_ (M = Mn, Fe, Co, Ni) by DFT+U calculations and analyzed their trends to find several unusual types of spin exchanges unknown from other types of spin exchanges known so far. 

(5) The contribution by Samir Matar and Jean Etourneau [6] examined the chemical bonding and electronic structures of interstitial boron suboxides Bi_12_O_2_X (X = B, C, N, O) using first-principles DFT calculations to find that Bi_12_O_2_X has unpaired electrons on X for X = C and N with moments of 1.9 and 1 μ_B_, respectively, in the ferromagnetic ground state. 

### 1.2. Contributions in the Area of Lithium-Ion Batteries

In the following, the first eight papers are invited contributions.

(1) The article by Julia H. Yang, Haegyeom Kim and Gerbrand Ceder [7] perfectly illustrates the philosophy of Goodenough concerning his pursuit to understand the structure–property relationship, rationalize experimental observations and improve the characteristics of a studied material or device. On the basis of DFT calculations, the authors evidence how the topology of layered structures impacts the electrochemical performances. In a more general way, the article demonstrates how crucial first-principles calculations are in accounting for the properties of materials and how they help to select new promising candidates for electrodes.

(2) Atsuo Yamada [8] provided a short review on polyanion positive electrode materials, which can generate high-voltage generation batteries with high-density energy. This paper gives a general overview of cell voltage monitoring vs. transition element electronic configuration. Currently, a challenging problem is to find cations other than those derived from Ni, Co and V, three transition elements that are still commonly used but are too expensive. As alternative possibilities, investigations were carried out with Fe^3+^/Fe^2+^ and Cr^4+^/Cr^3+^ redox couples, and sulfate and phosphate as the framework structure with Li^+^ or Na^+^ as mobile species. 

(3) High-voltage multi-electron reactions in alkali-ion batteries using vanadium phosphate positive electrodes are described by Edouard Boivin, Jean-Noël Chotard, Christian Masquelier and Laurence Croguennec [9]. They clearly showed how the vanadyl distortion affects the reversibility of the intercalation/de-intercalation mechanism, the voltage of the battery and the number of exchange electrons per transition metal, and how these parameters can be modified by controlling the local chemical environment of vanadium, i.e., by an appropriate change in the composition (e.g., fluorine/oxygen exchange) of the material.

(4) Lithium–sulfur batteries can provide a higher energy density than classical Li-ion batteries at a lower cost, and they are environmentally friendly. Here, Sang-Kyu Lee, Hun Kim, Sangin Bang, Seung-Taek Myung and Yang-Kook Sun [10] show how the addition of WO_3_ nanowires mixed with carbon nanotubes at the separator/cathode interface may improve the performance of such a device.

(5) Until recently, batteries and specifically positive electrodes were made from inorganic materials. Margaud Lécuyer, Marc Deschamps, Dominique Guyomard, Joël Gaubicher and Philippe Poizot [11] report the electrochemical performance of Li-based metal batteries involving indigo carmine, an insoluble organic salt, as the positive electrode. This opens the possibility toward inexpensive and renewable materials, a desired goal that future batteries must achieve.

(6) In this article devoted to a perovskite-type Li-ion conductor, Guowei Zhao, Kota Suzuki, Masaaki Hirayama and Ryoji Kanno [12] focus on a (Li_x_La_1−x/3_)ScO_3_ solid solution prepared under high pressure and doped with Ce^4+^ and Zr^4+^ or Nb^5+^ to achieve a high ionic conductivity. 

(7) This is another article devoted to a perovskite-type Li-ion conductor. Jinhua Hong, Shunsuke Kobayashi, Akihide Kuwabara, Yumi H. Ikuhara, Yasuyuki Fujiwara and Yuichi Ikuhara [13] studied (Li_x_La_1−x/3_)NbO_3_ to examine the structural defects, such as point defects and grain boundaries, that may significantly perturb the migration of Li^+^ ions. Controlling such structural defects is essential in constructing solid electrolyte batteries and their all solid-state variants. This study presents new very-high-resolution electron microcopy techniques.

(8) This contribution by Seona Kim, Guntae Kim and Arumugam Manthiram [14] is concerned with rechargeable metal–air batteries. As an example of cost-effective electrocatalysts with effective bifunctional activity for both oxygen reduction and oxygen evolution, they discussed a hybrid catalyst, Co_3_O_4_-infiltrated La_0.5_Sr_0.5_MnO_3-*δ*_, to demonstrate that hybrid catalysts are a promising approach for oxygen electrocatalysts for renewable and sustainable energy devices.

(9) In the last contribution [15], Jan L. Allen, Bria A. Crear, Rishav Choudhury, Michael J. Wang, Dat T. Tran, Lin Ma, Philip M. Piccoli, Jeff Sakamoto and Jeff Wolfenstine examined Li-stuffed spinels with a high conductivity of Li as a material for a fully structured all solid battery. Their work provides a useful concept for enabling a single-phase fully solid electrode without interphase impedance.

## Figures and Tables

**Figure 1:**
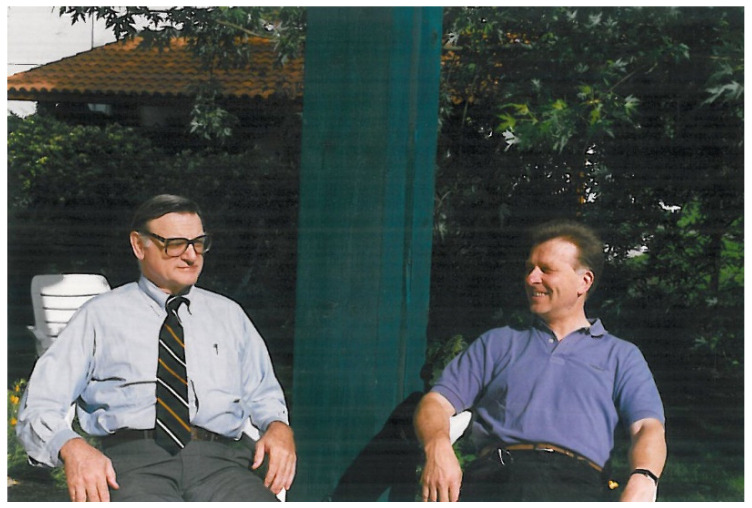
John Goodenough and Jean Etourneau at Jean’s in the summer of 1998.
